# Cardioprotective Effect of Ethanolic Leaf Extract of *Melissa Officinalis* L Against Regional Ischemia-Induced Arrhythmia and Heart Injury after Five Days of Reperfusion in Rats

**DOI:** 10.22037/ijpr.2019.1100761

**Published:** 2019

**Authors:** Mehrnoosh Sedighi, Mahdieh Faghihi, Mahmoud Rafieian-Kopaei, Bahram Rasoulian, Afshin Nazari

**Affiliations:** a *Razi Herbal Medicines Research Center Lorestan University of Medical Sciences Khorramabad, Iran. *; b *Cardiovascular Research Center, Shahid Rahimi Hospital, Lorestan University of Medical Sciences, Khoramabad, Iran.*; c *Department of Physiology, School of Medicine, Tehran University of Medical Sciences, Tehran, Iran. *; d *Medical Plants Research Center, Basic Health Sciences Institute, Shahrekord University of Medical Sciences, Shahrekord, Iran.*; e *Razi Herbal Medicines Research Center and Department of Physiology, Lorestan University of Medical Sciences, Khorramabad, Iran. *

**Keywords:** Melissa officinalis, Ischemia, Reperfusion injury, Heart, Rat

## Abstract

*Melissa officinalis* has antioxidant and anti-inflammatory activities and is used in various diseases. Aim of the study*:* We investigated the role of *M. officinalis* extract (MOE) against ischemia-induced arrhythmia and heart injury after five days of reperfusion in an *in-vivo* rat model of regional heart ischemia. The leaf extract of *M. officinalis* was standardized through HPLC analysis. Adult male Sprague-Dawley rats (n = 32) were subjected to 30 min of ischemia by occlusion of the left anterior descending coronary artery followed by 5 days of reperfusion. The rats (n = 8 in each group) were randomized to receive vehicle or *M. officinalis* as follows: group I served as saline control with ischemia, groups II, III and IV received different doses of MOE- (25, 50 and 100 mg/kg, respectively), by oral gavage daily for 14 days prior to ischemia. Administration of *M. officinalis* significantly improved ischemia/reperfusion (I/R)-induced myocardial dysfunction by reduction of infarct size, also, during the ischemic period, ventricular tachycardia, and ventricular ectopic beats episodes decreased as compared with that of the control group. Stabilized ST segment changes and QTc shortening increased the R and T wave amplitudes and the heart rate during ischemia. The extract also caused significant elevations in serum superoxide dismutase (SOD) activity as well as a significant decrease in serum cardiac troponin I (CTnI), lactate dehydrogenase (LDH), and malondialdehyde (MDA) levels, 5 days after reperfusion. MOE-100mg/kg was the effective dose. Cinamic acid (21.81 ± 1.26 mg/gr) was the main phenolic compound of plant sample. The ethanol extract of *M. officinalis *was observed to exhibit cardioprotective effects against I/R injury, probably due to antioxidant properties. The results indicate that MOE has antioxidant and cardio-protective effects against ischemia-induced arrhythmias and ischemia-reperfusion induced injury as was reflected by reduction of infarct size and cardiac injury biomarkers. These data support the potential uses of M. officinalis in the treatment of heart ischemia- reperfusion disorders and even developing new anti- arrhythmias drugs after further investigations.

## Introduction

Preventing issue damage following MI is accomplished through reperfusion of the ischemic myocardium (1). However, reperfusion in the heart is accompanied by generation of free radicals and induction of oxidative stress which play a substantial role in lipid peroxidation and tissue damage arising from I/R (2, 3)

Despite substantial advances in medicine, alternative and complementary medicine has nowadays grown for MI complications, in developed and developing countries. Attempts are also being made to get scientific evidence for traditionally reported plant derived drugs. This points to the importance of investigating natural agents used in folk medicine (4)

From the medicinal plants, *Melissa officinalis* is a promising plant for this purpose. It belongs to the genus Melissa (Lamiaceae family) (5) and is known as “Badranjboya” in Persian “Mountain balm” and “Lemon balm” in English (6). M. officinalis is a salient medicinal plant which is popular worldwide (7). It has been used in traditional medicine for various diseases including hypertension, migraines, menstrual problems, vertigo, fever, and epilepsy (8), bronchitis and asthma (9), bell′s palsy, paralysis and arthritis (10). 

Modern researches have shown its neuroprotective (11), anxiolytic (12, 13), antispasmodic (14), hepatoprotective, and anti hyperlipidemic properties (15). The beneficial effects of M. officinalis on heart palpitation relief  have been reported, recently in a double blind, randomized, placebo controlled trial based on its usage in TIM (16, 17). Also, the efficacy of Melissa officinalis in suppressing ventricular arrhythmias following ischemia-reperfusion of the heart was evaluated in comparison with amiodarone (18). However, no study has yet dealt with the effects of *M. officinalis* consumption on the heart in the presence of ischemic stressful conditions. This study was conducted to evaluate the pre-treatment effects of this herb on induced arrhythmias and heart injury after five days of ischemia reperfusion in an *in-vivo* rat model of regional heart ischemia. In addition to assessing the incidence of arrythemia during ischemia, we also evaluated the ECG changes within 5 days after reperfusion. Additionally, in this study, various parameters including hemodynamic changes, infarct size, and heart tissue antioxidant enzymes were investigated five days after reperfusion. The plant standardization was done through HPLC and antioxidant capacity measurement of the total extract of the plant. 

## Experimental

*General considerations*

The protocol of this study was approved by Razi Herbal Medicines Research Center and Medical Ethics Committee of Lorestan University of Medical Sciences (ethical code: LUMS.REC.1394.79)

Thirty two adult male Sprague-Dawley rats weighing 250-300 g were purchased from the Research Center of Lorestan Province. The animals were maintained in a temperature controlled room at 21–24 °C and 40–50% humidity, a 12h light/dark cycle with free access to food and water.

*Materials and preparation of plant extract*

*Melissa officinalis *samples were purchased from local groceries across Ilam, southwest of Iran in April-May and identified as *Melissa officinalis *by a botanist. All chemicals were obtained from Sigma-Aldrich (USA), unless otherwise stated.

Coarse powder from dried leaf of* Melissa officinalis *was extracted to exhaustion with ethanol (96%) using a soxhlet apparatus with ethanol solvent systems. The extract was dissolved in sterilized distilled water before oral administration to the experimental animals (18). 

*Determination of chemical composition *

The amount of cinamic acid in *M. officinalis* extract was assessed by High-performance liquid chromatography (HPLC) method. The HPLC consisted of a manual injector with a 10 μL sample loop, a quaternary pump (LC-10ATvp), and a C_18_ analytical column (Shimadzu, 5 μm particle size, 150×4.6 mm id; Japan). The effluent was monitored by UV-Vis detector (SPD-M10, Japan) at 280 nm. The HPLC mobile phase consisted of water (containing 0.1% v/v acetic acid) and acetonitrile (80/20, v/v) which were degassed by a Millipore vacuum pump (Shimadzu, Japan) prior usage. The flow rate was set at 1.0 mL/min.

*In-vitro antioxidant tests*

*Determination of total phenolic content*

The total phenolic content of *Melissa officinalis *extract was determined using the Foline Ciocalteu method as modified by Singleton & Lamuela-Raventos (19).

*Determination of total flavonoid content*

The total flavonoid content was determined using a colorimetric method described by Dewanto (20).

*Assay for total antioxidant activity*

*DPPH radical scavenging test*

Reduction of the stable free radical DPPH was determined with the aid of a modified version of the method described by Shimada. The result was expressed as a percentage of the absorbance of the control DPPH solution without test compounds (21).

*Surgical preparation*

Anesthesia was achieved in animals by administration of sodium thiopental (60 mg/kg body weight, i.p.), their chests were completely shaved and they were placed on operating table. During the surgery, the temperature of the rats′ bodies was maintained at 37±1 °C using a thermo-regulator (HARVARD USA). The rats′ necks were positioned in a way that tracheal intubation could be easily conducted on them. After intubation, the animals were ventilated at 60-70 breaths/min and tidal volume 1.5 mL/kg by an animal ventilator (HARVARD USA). To record ECG lead II, subcutaneous needle electrodes were used such that the negative electrode was subcutaneously connected to the right arm, the positive electrode to the left foot and the neutral electrode to the left arm. Then, an incision was made on the left fourth intercostal space of the chest so carefully that the left lung and heart were not injured. Afterwards, pericardium was slowly torn and silk thread 6-0 silk carefully passed through under LAD and fastened with a newly designed ligature apparatus (Bavanesh: A device useful for coronary ligation in rats, Elmbavaran Aftab Lorestan Company, Iran). Reperfusion was induced by dragging and releasing the thread of Bavanesh.

After completion of the ischemia and beginning of the reperfusion, the incision was closed, the tracheal tube was removed, and the rats were completely oxygenated so that they recovered. Two criteria, i.e. disorder in normal myocardial contraction and change in ST segment in ECG, were considered to approve induction of MI. After complete recovery, the rats were transferred to Animal House. The rats were scarified 5 days after reperfusion induction. Except for ECG, which was recorded during ischemia and first 60 minutes of reperfusion, other variables were examined at the end of the fifth day. Tetracycline ointment was used as topical antibiotic.

*Experimental protocol*

The rats were randomly assigned to 4 groups, each comprising 8 rats. Three groups were given *M. officinalis* extract (MOE) 50, 100 or 200 mg/kg/d and the control group was given distilled water. All treatments were oral. The rats were pretreated for 14 days and then subjected to 30 min regional heart ischemia and 5 days reperfusion. Blood samples (2 mL) were collected by cardiac puncture for serum enzyme assays.

*Hemodynamic functions*

Hemodynamic parameters such as systolic blood pressure (SAP), diastolic blood pressure (DAP), heart rate (HR), pulse pressure (PP), and mean arterial pressure (MAP) were recorded after five days of reperfusion.

**Figure 1:**
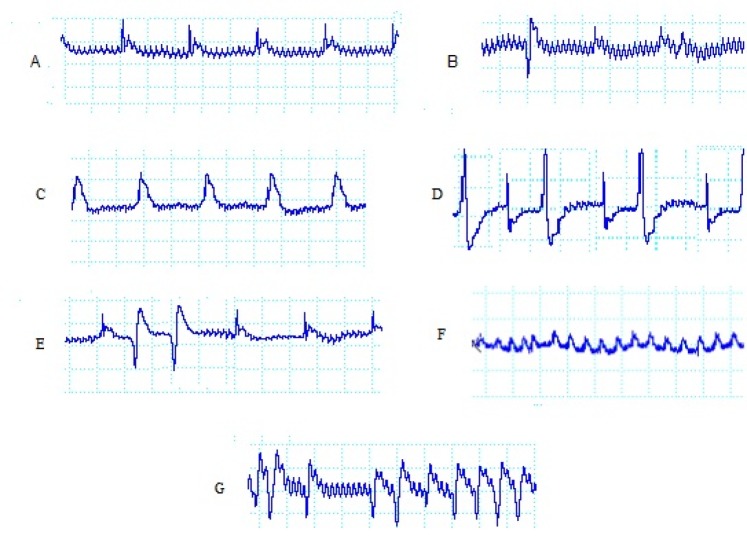
Examples of electrocardiogram recordings and definition of various changes and arrhythmias: During baseline (A), single VEBs (B), ST segment during ischemia (C), by-geminate(D), couplet (E), ventricular fibrillation (F) and ventricular tachycardia (G)

**Figure 2:**
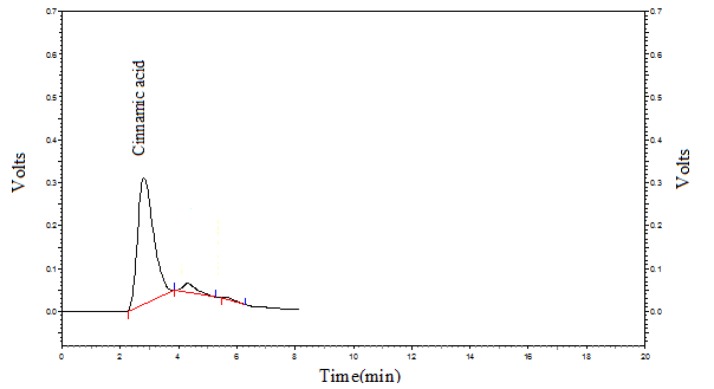
HPLC chromatographic pattern for determination of Cinnamic acid from extract of *Melissa officinalis *powder

**Figure 3:**
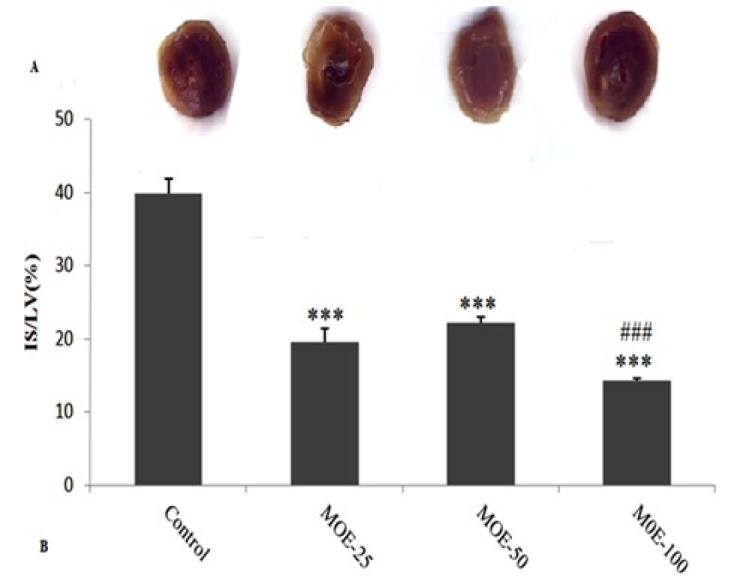
(A) The original pictures of TTC staining heart slices. (B) Myocardial infarct size (IS/LV %) in control and MOE (25, 50, 100) groups. Data are presented as mean ± SEM. ***P < 0.001 vs. control. ###*P *< 0.001 vs.MOE-50 group. MOE = *Melissa* *officinalis* extract-treated groups

**Figure 4:**
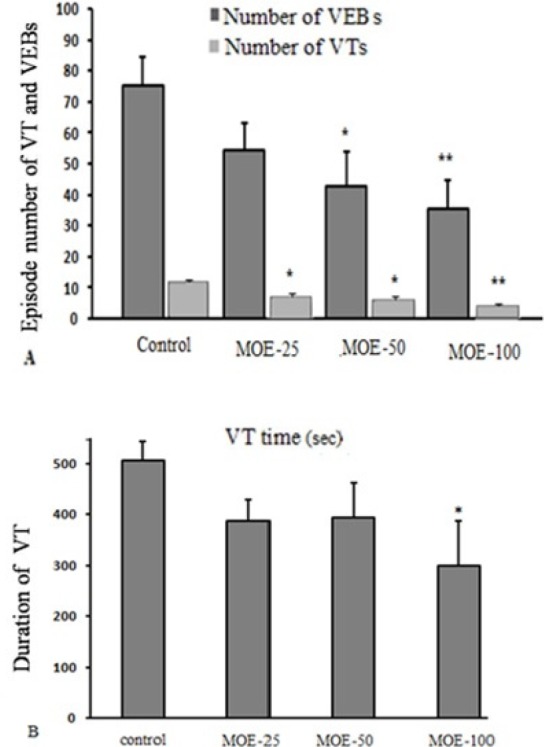
(A) Total number of VT (ventricular tachycardia) and VEB (ventricular ectopic beats) episodes during 30 min ischemia in different groups. (B) Total duration of VT episodes during 30 min ischemia in different groups. Data are presented as mean ± SEM.

*P < 0.05, **P < 0.01 vs. Control group. MOE = *Melissa officinalis *extract.

**Figure 5:**
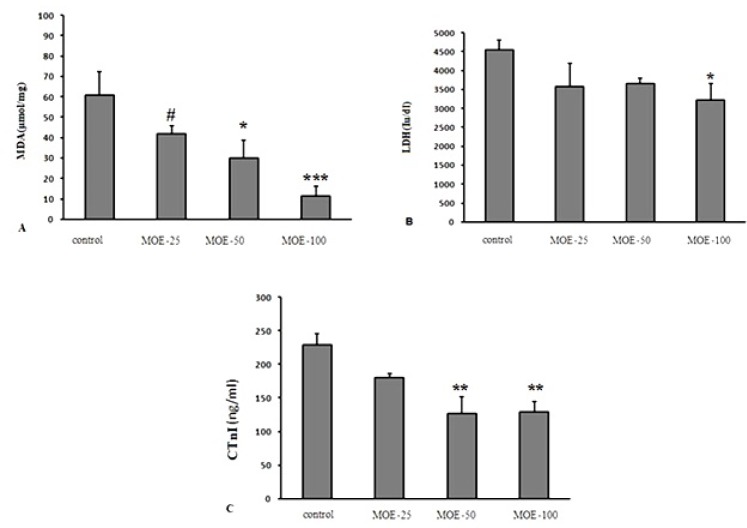
Effect of various treatments on serum levels of MDA (A), LDH (B) and CtnI (C) at 5 days after reperfusion in different groups. The values are expressed as mean±SEM; cTnI = Cardiac troponin I; LDH = lactate dehydrogenase; MDA = malondialdehyde. Data are presented as mean ± SEM. * Significant difference compared to the control group (*p *< 0.05); ** Significant difference compared to the control group (*p *< 0.01); *** Significant difference compared to the control group (*p *< 0.001); # Significant difference compared to the 100 mg/kg *M*. *officinalis *extract-treated group (*p *< 0.05)

**Table 1:** Changes in heart rate in various groups. The values are mean ± SEM; HR = heart rate (beats/min); (n: 8 in all groups)

**Groups**	**Baseline**	**End of ischemia 30'**	**End of reperfusion 60'**
Control	241.5±2.46	228.87±3.34	231.87±4.65
MOE-25	219±13.33	269.87±14.93*	194.37±14**$$**
MOE-50	196±14.38	240.25±14.23	170±14.39 **#$$**
MOE-100	265.5±39.80	312.87±25.06##&	214.75±15.7**$**

* Significant difference compared to baseline (*p *< 0.05); $ Significant difference compared to the end of ischemic period (*p *< 0.05); $$ Significant difference compared to the end of ischemic period (*p *< 0.01); # Significant difference compared to control group (*p *< 0.05). ## Significant difference compared to control group (*p *< 0.01). & Significant difference compared to MOE-50 (*p *< 0.05).

**Table 2:** Hemodynamic parameters at 5 days after reperfusion in different groups (n: 8 in all groups). The values are mean ± SEM; MAP (mean arterial pressure), SAP and DAP (systolic and diastolic arterial pressure) and PP (pulse pressure)

**Groups**	**(MAP, mm/gH)**	**(SAP, mm/gH)**	**(DAP, mm/gH)**	**(PP, mm/gH)**
Control	84.12±6.56	126.5±11.93	80±6.81	26±5.09
CI-50	74.16±2.16	86.57±3.01**	49.28±4.64**	17.98±3.51
CI-100	80.3±8.16	92.26±6.41**	58.57±3.04*	14.39±3.29*
CI-200	80.62±3.81	312.87±25.06##&	65±7.82	12.40±2.01*

*Significant difference compared to the control group (*p *< 0.05). ** Significant difference compared to the control group (*p *< 0.01).

**Table 3:** Changes in ECG parameters of various groups

**Time**	**Variable**	**Control**	**MOE-25**	**MOE-50**	**MOE-100**
Baseline	QTC(ms)	242.25±24.86	206.12±23.88	122.37 ±10##	179.87±26.98
	QRS(μV)	17±0.59	17±0.65	17.25±0.83	13.75±0.25## ££
	R(μV)	524.62±49.83	466.62±24.53	421.12±41.97	426.62±39.85
	T(μV)	267.87±48.16	170.75±14.49	200.62±28.04	231.5±13.13
	ST(μV)	252.5±20.21	194.5±27.06	178.62±24.54	129.12±6.05##
End of ischemia 30	QTC(ms)	279.75±21.89	119.37 ±4.50*###	150.87±13.37 ###	103.75±3.25*###
	QRS(μV)	19.62±1.01	15.62±0.41##	16±0.59 ##	13.87±0.39 ###
	R(μV)	612.87±46.82	523.5±21.05	474±38.37	618.25±34.57**£
	T(μV)	233±25.48	217.25±24.32 £	252.25±49.49	365.5±35.72*
	ST(μV)	319.7±44.73	254.37±22.32	141.75±12.71##	151.75±32.92##
End of reperfusion 60	QTC(ms)	250.62±20.07	201.37±26.68$	116.5±9.55###£	152.75±16.59 ##
	QRS(μV)	17.75±0.86	16.12±0.63	16.25±1.43	13.87±0.29 #
	R(μV)	530.37±72.99	370.25±33.65*$$	380.87±37.74	449.75±37.43$
	T(μV)	250.75±22.51	149.5±12.69$	187. ±36.70	232±35.12 $
	ST(μV)	314±29.42	184.87±27.57 ##	223.25±19.90 # $	127.5±7.76 ### &

The values are mean ± SEM.

* Significant difference compared to baseline (*p *< 0.05); ** significant difference compared to the baseline (*p *< 0.01); ## significant difference compared to control group at corresponding interval (*p *< 0.01); ### significant difference compared to the control group at corresponding interval (*p *< 0.001); $ significant difference compared to ischemia duration (*p *< 0.05); $$ significant difference compared to ischemia duration (*p *< 0.01); £ Significant difference compared to ischemia duration in 50 mg/kg *Melissa officinalis *group (*p *< 0.05);

££ Significant difference compared to ischemia duration in 25 and 50 mg/kg *Melissa officinalis *groups (*p *< 0.01); & significant difference in reperfusion duration compared to 50 mg/kg *Melissa officinalis *extract-treated group in the corresponding interval (*p *< 0.05).

**Table 4:** Effect of various treatment on serum superoxide dismutase (SOD), catalase (CAT) and glutathione peroxidase (GPX) 5 days after reperfusion

**Groups**	**SOD (unit/mg)**	**CAT(unit/mg)**	**GPX(unit/mg)**
Control	40±5.24	133.52±24.29	686.17±42.15
MOE-25	41.42±14.95	119.29±18.93	727.13±18.45
MOE-50	37.5±15.86	111.77±14.95	811.76±24.17
MOE-100	104.28±16.08*	157.45±12.50	904.69± 15.57

The values are mean ± SEM of 8 animals in each group. * Significant difference compared to control, MOE-25and 50 groups (*p *< 0.05).

*Determination of infarct size*

At the end of the experiment, the heart was excised and both atria and the roots of the great vessels were removed. The heart was frozen overnight and then cut into slices of 2-mm-thick. All slices were incubated with a 1% solution of 2,3,5-triphenyltetrazolium chloride (TTC, in 0.1 M phosphate buffer, pH 7.4) stain for 20 min at 37 ºC to visualize the infarct area. Then, they were fixed in 10% formalin. Both surfaces of each section were scanned using PhotoShop program (Adobe Systems, version 7.0). Infarct size percent was expressed as a percentage of the left ventricles (IS/LV).

*Determination of arrhythmias and electrocardiogram parameters*

During the 30-min ischemic period, ventricular arrhythmias were evaluated. Ventricular ectopic beats (VEBs) were defined as identifiable premature QRS complexes. Ventricular tachycardia (VT) was defined as a run of four or more ventricular premature beats and ventricular fibrillation (VF) was defined as a signal for which individual QRS deflections could no longer be distinguished from each other and for which a rate could no longer be measured. A number of original ECG recordings as examples of the so defined arrhythmias are illustrated in Figure. 1. 

The incidence, onset time, and duration of arrhythmias were assigned to identify arrhythmias severity according to a previously defined scoring system (22).

The QRS interval duration, corrected QT interval (QTc), T and R amplitude and ST-segment elevation were recorded at baseline, end of 30 min ischemia and 60 min of reperfusion. The QRS interval was analyzed. The QT interval was measured starting from the onset of the QRS complex until the end of the T wave. QTc was obtained using Bazett′s formula (QTc = QT/√RR) (23).

*Determination of MDA, LDH, CTNI, CAT, SOD and GPX in the serum*

Heparinized blood samples were collected at the end of five days of reperfusion and centrifuged at 5000 rpm, for 15 min. Plasma was removed and stored at −70 ºC until the time of assessments. Lactate dehydrogenase (LDH) activity and cardiac Troponin I (CTNI) levels were analyzed using commercial kits (Pars Azmoon, Iran and Zellbio, Germany) by employing an Autoanalyzer (Roche Hitachi Modular DP Systems, Mannheim, Germany). Malondialdehyde (MDA) content of samples, as an index of lipid peroxidation, was determined spectrophotometrically using a modification of the assay described by Schuh *et al*. Superoxide dismutase (SOD), glutathione peroxidase (GPX), and catalase (CAT) activity levels were measured spectrophotometrically using diagnostic kits according to the manufacturers′ instructions (24, 25).

*Statistical analysis*

Statistical analysis of arterial hemodynamic parameters, HR, and ECG parameters within the groups were performed with repeated measures ANOVA followed by the Tukey’s test. Other parameters were determined by one-way ANOVA followed by the Tukey’s test. All data were expressed as mean ± SEM. Statistical significance was defined as *P* < 0.05.

## Results

*Radical scavenging activity, flavonoids and total phenolic contents of M. officinalis *leaf extract the antioxidant activity of the extract was 60.51% of the Rutin. The flavonoid and total phenolic compounds were, respectively, 7.80 and 26.68 mg/g of dried extract.

*HPLC analysis of M. officinalis leaf extract*

HPLC analysis of the plant extract was done to determine Cinnamic acid content of the *M. officinalis* leaf extract. Cinnamic acid level was 21.81 ± 1.26 mg/gr. Figure 2 shows the Cinnamic acid chromatogram of the sample.

*Hemodynamic functions*

Table 1 demonstrates the time course of heart rate (HR) during the experiment. There were no significant differences among groups at baseline (before ischemia). Heart rate differences in control group at baseline, end of ischemia and end of 60 min of reperfusion were not statistically significant. In overall, MOE administration, led to an increased HR at the end of ischemic period compared to baseline. 

After 60 min of reperfusion, again HR decreased compared to values of the end of ischemic period. These low values of the HR in MOE-treated groups were even lower than baseline rates, although this was not statistically significant. Details are demonstrated in table 1. 

Table 2 demonstrates the time course of mean arterial pressure (MAP), systolic arterial pressure (SAP), diastolic arterial pressure (DAP), and pulse pressure (PP) during the experiments at 5 days after reperfusion. SAP significantly decreased in treatment MOE-25, 50 and 100 groups, DAP significantly decreased in treatment MOE-25 and 50 groups and PP significantly decreased in treatment MOE-50 and 100 groups as compared with their controls.

*Infarct size measurements*

Figure 3A shows the original pictures of the stained hearts by TTC. The infarct size (IS/LV) is shown in Figure. 3B. Infarct size was 39.85 ± 2.05% in control group, whereas administration of different doses of MOE (25, 50 and 100) significantly reduced infarct sizes to 19.53 ± 1.93%, 22.17 ± 0.79%, and 14.28 ± 0.28%, respectively. The infarct size was significantly lower in MOE-100 group than in MOE-50 group (*p *< 0.01).

*Ventricular arrhythmias during ischemia*

Figure. 4A shows the total number of VEBs and VTs. The mean total number of VEB episodes during 30 min of ischemia in MOE-50 and 100 groups markedly reduced, compared to the control group (42.62 ± 11.23 and 35.37 ± 9.3 vs. 76.25 ± 7.15, respectively). Also, treatment with *M. officinalis* extract in MOE-25, 50 and 100 groups, significantly reduced the mean total number of VT episodes, compared to the control group. 

Figure 4B shows mean total duration of all VT episodes. Compared to the control rats, duration of VTs throughout 30 min of ischemia was significantly reduced by administration of *M. officinalis *in MOE-100 group (299.16 ± 87.45 s in MOE-100 group vs. 507 ± 36.93 s in control group). In this regard, MOE-25 and 50 groups had no significant effect compared to the control rats.

*Other ECG parameters*

The ECG analysis showed noticeable variation, including QTc interval shortening in treatment MOE-(25, 100) groups at the end of ischemia period compared with its baseline. Repolarization voltage (T-wave) significantly increased in treatment MOE-100 group at end of ischemia period compared with its baseline and significantly decreased in MOE-25 and 100 groups at the end of reperfusion period compared with its baseline. R-wave voltage significantly increased in treatment MOE-100 group at the end of ischemia period compared with its baseline and significantly decreased in MOE-25 group at the end of reperfusion period compared with its baseline and ischemia (Table 3).

Intergroup comparison showed that at baseline, QTc was significantly shorter in MOE-50 group compared to control group and QRS interval was significantly shorter in MOE-100 group compared to MOE-25, 50, and control groups. The mean value of ST segment voltage was significantly lower in MOE-100 group as compared to the control group.

At the end of ischemic period, QTc and QRS significantly decreased in treatment MOE-(25, 50 and 100) groups as compared with the control group. R-wave amplitude significantly increased in MOE-100 group compared to MOE-50. T-wave amplitude significantly increased in MOE-100 group as compared with MOE-25 group. Finally, administration of *M. officinalis*in MOE-(50 and 100) groups decreased the ST segment voltage changes compared with control group.

At end of reperfusion period, QTc significantly decreased in treatment MOE-(50, 100) groups compared with the control group, QRS was significantly decreased in treatment MOE-100 group compared to the control group and ST segment voltage was significantly decreased in MOE- (25, 50 and 100) groups as compared with the control group and decreased in MOE-100 compared with MOE-50 group (Table 3).

*Biochemical analysis*

*Antioxidant enzymes activities*

CAT, SOD, and GPX activities were examined 5 days after reperfusion in serum samples in various experimental groups. As shown in Table 4, SOD activity significantly increased in the MOE-100 group compared to MOE-25, 50 and control groups (*p *< 0.05).

*CTNI level and LDH activity and lipid peroxidation level*

Serum MDA level in MOE-(50 and 100) groups significantly declined compared to the control group and it was significantly lower in MOE-100 group compared to MOE-25 (Figure.5A). Also, administration of* M. officinalis *extract in MOE-100 group significantly prevented LDH activity elevation. Serum CtnI level, as a biomarker of myocardial injury was lower in MOE-50 and 100 groups compared to control group, 5 days after reperfusion (Figure.5 B & C).

## Discussion

In this study the ethanolic extract of *M. officinalis *was observed to exhibit cardio-protective effect against I/R injury. This was reflected by a reduction in infarct size, episodes of VTs and VEBs and total duration of VTs, decrease in ST segment changes and QTc and QRS shortening. Also the extract administration led to increased serum SOD activity and decreased serum CtnI, LDH, and MDA levels 5 days after reperfusion.

Since more than two decades ago, the role of reactive oxygen species in many cardiovascular diseases has become increasingly apparent. Under normal conditions there is a balance between the formation of oxygen-free radicals and the amount of anti-oxidants. This steady-state condition may be interrupted in some pathophysiologic conditions -like an ischemic insult and subsequent reperfusion- because of the excessive production of free radicals and/or decrease in anti-oxidants (26-29). A significant amount of evidence is present in the literature to support the role of oxygen free radicals in pathogenesis of myocardial ischemia reperfusion injury (27, 30) This receives further support from this fact that many free radical scavengers and antioxidants are capable of ameliorating ischemia-reperfusion injury (31). Total phenolic compounds and flavonoid contents of dried *M. officinalis* leaf extract used in present study showed relatively high amounts (about 27 and 8 mg/g, respectively). Furthermore, there is an inverse association between flavonoid intakes and coronary heart disease mortality (32, 33). Some clinical studies showed that flavonoids might reduce mortality from coronary heart disease (34). The most important property of the flavonoids is their antioxidant activity that could be due to scavenging of free radicals, interfering with inducible nitric-oxide synthase activity and inhibition of xanthine-oxidase (32). This study and previous studies have confirmed that *M. officinalis L* possesses high level of antioxidant activity through its chemical compounds including high amounts of phenolic contents and flavonoids. It seems that antioxidant properties, especially phenolic contents of the extract may have important role in preventing I/R induced injuries such as arrhythmias and infarction (35, 36). HPLC analysis of the administrated extract showed relatively high amounts of cinnamic acid as a main phenolic compound of the plant sample. Cinnamic acid derivatives in the plants are naturally occurring components found in a wide variety of flowers (37), vegetables, and fruits (35). Cinnamic acid has certain pharmacological properties including anti-inflammatory, anti-oxidative, anti-tumoral, anti-hypertensive, and anti-hyperlipidemic activities. Also, it is able to minimize the oxidation of low-density lipoproteins (LDL) (38-40).* M. officinalis* extract in present study may be related at least partially to the above-mentioned antioxidant effects of this herbal extract. This is supported by the fact that there are higher antioxidant enzymes, SOD activities in serum of rats receiving *M. officinalis* (100mg/kg) extract administration and also lower serum MDA levels in this group of rats. It should be noted that the serum MDA level has been used as an indicator of tissue damage caused by *in-vivo* free oxygen radicals (41, 42). In one previous study, the aqueous extract of *M. officinalis* significantly reduced the heart rate in an isolated heart model in rats (17). In the present study, the mean heart rate of animals which received 14 days oral administration of *M. officinalis* methanolic extract were not different from control group at baseline which is in consistent with the mentioned previous study. Even, in MOE-treated groups, there was an increased HR during the ischemic period which was transient and decreased to its baseline or even lower values, 60 min after ischemia. Historically lemon balm has been said to possess sedative/tranquilizing, anti-gas, fever-reducing, antibacterial, spasmolytic (43), hypotensive, memory-enhancing, menstrual-inducing, thyroid-related effects, and antiviral, antioxidant, antifungal, antiparasitic and antispasmolytic activities. It is also used in flatulence, asthma, bronchitis, amenorrhea, cardiac failure, arrhythmias, ulcers and wounds (44, 45). Also M. officinalis has been effective in heart palpitation relief in a recent clinical trial designed based on its traditional use in traditional medicine (14). This study is the first one which shows directly its potent cardio-protective actions and anti-arithmetic effects. Overall, in modern scientific research, less attention has been devoted to the cardiac effects of *M. officinalis*. A mild antiarrhythmic effect of *M. officinalis* was shown in another previous study (46), but in our study, *M. officinalis* methanolic leaf extract showed considerable dose-dependent antiarrhythmic effects during the ischemia period. The duration of action potential (APD) is determined mainly by the duration of repolarization, which is influenced by termination of calcium influx and potassium efflux. So, either factor inhibiting calcium influx or promoting potassium efflux may shorten APD (47, 48). So, developing anti-arithmetic drugs without QT prolongation effects is one of the main targets for many researchers. As was mentioned, the anti-arithmetic properties of *M. officinalis* extract in present study was associated with a significant decrease of QTc interval. Administration of *M. officinalis* significantly reduced the levels of injury markers including cTnI and LDH. Especially, cTnI, an excellent serum marker for detecting myocardial injury was significantly reduced by *M. officinalis* extract administration, supporting our data about lower infarct size in this group.

## Conclusion

The present study revealed that the ethanolic leaf extract of *M. officinalis* exhibits cardio-protective effects against ischemia-induced arrhythmias and ischemia-reperfusion induced injury as was reflected by a reduction in infarct size and cardiac injury biomarkers. The antioxidant properties of *M. officinalis* extracts which are partially attributable to the ability of its phenolic constituents to quench reactive oxygen species are thought to contribute in these protective properties. These data support the potential uses of *M. officinalis* in the treatment of heart ischemia- reperfusion disorders and even developing new anti- arrhythmias drugs after further investigations.
